# Multiregional Fractal Analysis of Mandibular Trabecular Architecture for Sex Estimation: A Panoramic Radiographic Study

**DOI:** 10.2174/0115734056521694260805045218

**Published:** 2026-08-21

**Authors:** Seda Akı Başer, Mehmet Çolak, Ezgi Katı

**Affiliations:** 1 Department of Oral and Dentomaxillofacial Radiology, Faculty of Dentistry, Dicle University, Sur, Diyarbakır, Türkiye

**Keywords:** Sex determination, Panoramic radiography, Mandible, Image processing, Bone remodeling, Mandibular condyle

## Abstract

**Background/Objective:**

Fractal Analysis (FA) is a mathematical method used to quantify complex self-similar structures and provides a numerical representation of trabecular bone microarchitecture on radiographic images. This study aimed to evaluate region-specific sex differences in Fractal Dimension (FD) and assess the forensic applicability of FA for sex estimation.

**Materials and Methods:**

This prospective study analyzed 130 digital panoramic radiographs from a systemically healthy cohort (65 males, 65 females; aged 20–40 years). Six bilateral regions of interest (50 × 50 pixels), representing the condylar, angular, and interdental trabecular regions, were selected, yielding 780 ROIs. FD values were calculated using ImageJ with a standardized box-counting method. Sex-related differences were analyzed using appropriate statistical tests and binary logistic regression.

**Results:**

Intra-observer reliability was good to excellent (ICC: 0.767–0.879). Bilateral analysis showed significant sex-related differences in the left condylar (*p* < 0.001) and left interdental regions (*p* = 0.048). After bilateral averaging, only the condylar region remained significant. Females showed lower mean condylar FD values than males (1.381 ± 0.094 *vs*. 1.423 ± 0.091; *p* = 0.012). Logistic regression identified condylar FD as a significant predictor of sex (OR = 0.61, 95% CI: 0.41–0.91; *p* = 0.014), although discrimination was limited (AUC = 0.628; accuracy = 58.5%).

**Discussion:**

Sex-related differences in mandibular trabecular architecture appeared to vary across anatomical regions, with the condylar region showing the most consistent discriminatory ability. This finding may be related to the condyle’s greater susceptibility to biomechanical adaptation and hormonal influences, both of which can affect trabecular organization.

**Conclusion:**

FA of panoramic radiographs may serve as a useful adjunct in forensic sex estimation. However, its moderate discriminative performance suggests that it should be used in combination with complementary analytical methods rather than as an independent diagnostic tool.

## 1 INTRODUCTION

Forensic human identification constitutes a cornerstone of medico-legal investigations, particularly in scenarios involving decomposed, fragmented, or otherwise unrecognizable human remains. The reconstruction of a biological profile typically encompasses the systematic determination of sex, age, ancestry, and stature [1]. Among these core parameters, sex estimation is universally regarded as the pivotal initial step, as it substantially narrows the pool of prospective identities and strategically guides subsequent identification protocols [2]. Although conventional methods for sex estimation remain fundamental to forensic practice, continuous efforts are underway to refine existing techniques and develop novel methodologies to improve their accuracy and reproducibility [3].

The pelvis is considered the most reliable skeletal structure for sex estimation [4]; however, it is frequently unavailable or severely damaged in forensic contexts. Under such conditions, craniofacial structures provide important supplementary information, with the mandible representing a particularly valuable element due to its dense cortical structure and resistance to post-mortem degradation [5, 6].

Despite the availability of morphological, metric, and molecular methods for sex estimation, each approach has inherent limitations. Morphological assessments are subjective and dependent on examiner experience, while metric analyses require well-preserved skeletal material and population-specific standards. Molecular techniques, although highly accurate, are often constrained by cost, infrastructure requirements, and DNA degradation in forensic settings [3, 7].

Panoramic radiography is routinely used in contemporary dental practice as a foundational diagnostic tool, offering a comprehensive, bilateral overview of maxillofacial architectures with relatively low radiation exposure and minimal cost. The widespread clinical utilization and archival longevity of panoramic radiographs render them highly valuable retrospective repositories for forensic inquiries [8]. Beyond their conventional diagnostic role, a recently reported image analysis method demonstrated that substantially more quantitative information can be extracted from two-dimensional radiographs by quantifying X-ray attenuation, enabling reliable estimation of cortical and trabecular bone thickness characteristics [9].

Among these techniques, FA has emerged as a reliable method for quantifying trabecular complexity. Based on non-Euclidean geometry, FA enables evaluation of irregular, self-similar trabecular patterns that cannot be captured by conventional linear measurements. The derived Fractal Dimension (FD) reflects trabecular complexity and organization, with higher values indicating more complex and structured bone architecture [10]. Previous studies have primarily focused on the use of FA for evaluating systemic bone alterations and trabecular changes associated with various pathological conditions [11, 12]. However, evidence regarding its direct forensic application for sex estimation remains relatively limited [13, 14]. Previous investigations generally evaluated limited number of mandibular regions or focused on single-region analyses, leaving the comparative diagnostic contribution of multiple mandibular subregions insufficiently clarified. Therefore, the present study aimed to evaluate the effectiveness of region-specific FA of mandibular trabecular bone on panoramic radiographs for sex estimation in a Turkish population. It was hypothesized that certain mandibular regions would demonstrate significant sex-related differences in FD values and contribute to sex prediction.

## 2 MATERIALS AND METHODS

### 2.1 Ethics Approval and Consent to Participate

The study protocol was approved by the Clinical Research Ethics Committee of Dicle University on 30 December 2024 (Protocol No: 2024-33). All study procedures were carried out in accordance with the principles outlined in the Declaration of Helsinki. Written informed consent was obtained from all participants before inclusion in the study.

### 2.2 Study Population and Design

This prospective radiographic study included panoramic images obtained from 130 adults (65 males and 65 females) aged 20–40 years who were considered systemically healthy according to medical records and self-reported medical history. This age range was selected to minimize age-related trabecular alterations. The radiographs were collected from individuals who attended the Department of Oral and Maxillofacial Radiology, Faculty of Dentistry, Dicle University between 2024 and 2025. Reporting of sex and gender followed the Sex and Gender Equity in Research (SAGER) guidelines [15]. Biological sex (male/female), as recorded in the institutional medical records, was used as the study variable and accepted as the reference standard for all analyses. Gender-related variables were not collected because the objective of the study was to investigate biological sex differences in mandibular trabecular architecture.

Only high-quality radiographs demonstrating adequate visualization of the mandibular condyle, cortical outlines, and adjacent anatomical landmarks were included to ensure consistency in Region of Interest (ROI) selection and fractal measurements. Images affected by motion artifacts, distortion, overlapping structures, or inadequate trabecular visualization were excluded. In contrast to previous studies focusing on a limited number of anatomical sites, the present investigation adopted a bilateral multi-regional approach to explore possible regional variations in mandibular trabecular complexity associated with sex differences.

### 2.3 Power Analysis

An *a priori* sample size calculation was performed using G*Power software (version 3.1.9.7 for Windows; Heinrich-Heine-Universität Düsseldorf, Düsseldorf, Germany). The calculation was conducted using the logistic-regression option under the z-test family, with a two-tailed significance level of 0.05, statistical power of 95%, and an expected odds ratio of 2.674. This odds ratio was derived from previously reported ROI 5 data by Sindeaux *et al.* [16], which was considered the most comparable available evidence for mandibular fractal-dimension-based sex-related differences. The minimum required sample size was calculated to be 129 participants; therefore, 130 individuals were included in the final sample.

### 2.4 Inclusion and Exclusion Criteria

Individuals aged between 20 and 40 years without systemic disorders known to affect bone metabolism, including Paget’s disease, rheumatoid arthritis, and hyperparathyroidism, were eligible for inclusion. Additional inclusion criteria included the presence of complete maxillary and mandibular dentition excluding third molars, absence of bruxism, and no previous or ongoing orthodontic treatment history.

Exclusion criteria comprised mandibular fractures, pathological bone lesions such as cysts or tumors, orthodontic appliances, and dental implants. Individuals receiving medications known to affect bone metabolism or bone remodeling, including corticosteroids, bisphosphonates, and hormone-related therapies, were also excluded. In addition, radiographs presenting trabecular alterations within the ROIs or insufficient image quality, including elongation, blurring, distortion, or anatomical superimposition that could interfere with digital image processing, were excluded.

### 2.5 Dental Panoramic Radiographs

All panoramic images were obtained using the same panoramic imaging system (ProMax^®^, Planmeca Oy, Helsinki, Finland) to ensure methodological standardization. Image acquisition was performed by a single experienced radiology technician under standardized exposure conditions (66 kVp, 6 mA, 16 s) according to manufacturer recommendations for patient positioning and image acquisition.

### 2.6 Image Display Conditions

Radiographic evaluations were performed using a 21.3-inch color LCD monitor (RadiForce MX217, EIZO Corporation) under standardized viewing conditions.

### 2.7 Fractal Analysis Procedure

Panoramic radiographs included in the analysis were exported from the PACS software (PacsViewer^®^, Metasoft, Eskişehir, Türkiye) in high-resolution JPEG format and converted to TIFF format for standardized image processing. The radiographs had a resolution of 300 dpi (2943 × 1435 pixels) with a grayscale depth of 32 bits.

ROIs, each measuring 50 × 50 pixels, were selected bilaterally from mandibular trabecular bone areas. Six ROIs (three regions bilaterally) were positioned on each participant and corresponded to the condylar, angular, and interdental regions. In the condylar and angular areas, ROIs were centered within the trabecular bone of the relevant anatomical structure. In the interdental region, the ROI was placed between the mandibular second premolar and first molar. Care was taken to avoid cortical bone, root structures, and overlapping anatomical landmarks during ROI placement. Representative ROI localization is presented in Fig. (**1**).

FA was performed using ImageJ software (version 1.54g; National Institutes of Health, Bethesda, MD, USA) according to previously described image-processing protocols [13]. Initially, each ROI was selected (Fig. **1a**), duplicated (Fig. **1b**), and subjected to Gaussian blurring (sigma = 35) to minimize large-scale brightness variations (Fig. **1c**). The blurred image was subsequently subtracted from the original image (Fig. **1d**), after which grayscale normalization was applied by adding a constant pixel value of 128 to the intensity values of the resulting image (Fig. **1e**). The processed image was converted into binary format to distinguish trabecular structures from marrow spaces (Fig. **1f**). Morphological erosion (Fig. **1g**) and dilation (Fig. **1h**) procedures were subsequently applied to reduce image noise. Following image inversion (Fig. **1i**) and skeletonization (Fig. **1j**), FD values were calculated using the box counting algorithm with multiple box sizes ranging from 2 to 64 pixels.

To ensure methodological consistency and measurement reliability, a single investigator (SAB) underwent training and calibration procedures before the analysis. During all measurements, the examiner was blinded to the sex of the individuals in order to minimize potential observer bias.

### 2.8 Intra-observer Reliability

To evaluate intra-observer reliability, FD measurements obtained from 20% of the randomly selected radiographs were repeated by the same investigator after a one-month interval under identical conditions. Intraclass Correlation Coefficient (ICC) analysis was performed to assess measurement reproducibility.

### 2.9 Statistical Analysis

All statistical analyses were performed using R software (version 4.4.1). Normality of data distribution was evaluated using the Kolmogorov–Smirnov and Shapiro–Wilk tests. For bilateral comparisons, paired samples t-tests were applied for normally distributed variables, whereas Wilcoxon signed-rank tests were used for non-normally distributed data. Comparisons between male and female groups were performed using the independent samples t-test or Mann–Whitney U test depending on data distribution characteristics. To investigate the predictive capability of region-specific FD measurements for sex estimation, binary logistic regression analysis was conducted, and OR with 95% Confidence Intervals (CI) was calculated. Intra-observer reliability was assessed using ICC analysis. Quantitative data were expressed as mean ± standard deviation or median (minimum–maximum) values according to distribution characteristics. Statistical significance was accepted at *p* < 0.05.

## 3 RESULTS

The study population consisted of 130 participants (65 females and 65 males) aged between 20 and 40 years. The mean age was 24.74 ± 5.11 years for females and 24.74 ± 5.59 years for males. Six ROIs were defined for each participant, yielding a total of 780 ROIs included in the analysis. ICC values ranged from 0.767 (good agreement) to 0.879 (excellent agreement) across ROIs. Sex-based comparisons demonstrated statistically significant differences in FD values within the left condylar region (*p* < 0.001) and left interdental region (*p* = 0.048). In the left condylar region, FD values were lower in females than in males, whereas in the left interdental region, males exhibited higher FD values than females. No statistically significant sex-related differences were detected in the remaining ROIs (Table **1**). After averaging the right and left measurements for each anatomical site, only the condylar region showed a significant sex-related difference. Females had lower average condylar FD values than males (1.381 ± 0.094 *vs*. 1.423 ± 0.091; mean difference = −0.042; Cohen’s d = −0.45; *p* = 0.012), while the averaged angular and interdental regions were not significant. In logistic regression, average condylar FD remained a significant predictor of sex (OR per 0.1 FD increase = 0.61, 95% CI: 0.41–0.91; *p* = 0.014). The model showed limited discriminatory performance, with AUC = 0.628, accuracy = 58.5%, sensitivity = 53.8%, specificity = 63.1%, and Hosmer–Lemeshow *p* = 0.692 (Tables **2** and **3**).

## 4 DISCUSSION

The present study investigated sex-related differences in mandibular trabecular architecture using FA of panoramic radiographs. To our knowledge, this is among the few studies to evaluate multiregional mandibular FD measurements for sex estimation in a Turkish population. Our findings indicate that sexual dimorphism in mandibular trabecular bone is not uniformly distributed across anatomical regions but is site-dependent. In the initial bilateral analysis, statistically significant differences were observed in the left condylar and left interdental regions, whereas the remaining ROIs showed no significant variation. After bilateral averaging was performed to reduce side-dependent variability and provide a more anatomically representative assessment, only the condylar region remained significantly associated with sex, whereas interdental regions were no longer significant. This shift in statistical significance suggests that the initially observed interdental differences may not represent stable sex-related anatomical patterns but instead reflect localized functional adaptation or side-specific variability. The loss of significance after averaging supports the possibility that these findings were influenced by factors such as chewing-side dominance, occlusal asymmetry, postural differences, or stochastic sample-specific variation rather than true biological sexual dimorphism. Since masticatory dominance and side preference were not directly assessed in the present study, these findings should be interpreted with caution, as they may represent potential confounding factors. Unlike the interdental region, the condylar region remained significantly associated with sex even after bilateral averaging, underscoring its potential biological relevance and suggesting that the observed differences likely reflect true sexual dimorphism rather than measurement variability. Given its persistent discriminatory capacity, the mandibular condyle may represent the most robust and reliable anatomical region for evaluating sexual dimorphism in trabecular bone microarchitecture and may provide superior utility for sex estimation in forensic and dentomaxillofacial radiology. These findings are partially consistent with previous studies reporting regional variation in FD values between sexes. Çıtır *et al*. reported significant variations in the condylar and mandibular angle regions [17]. Similarly, Güleç *et al*. [18] identified sex-related differences in the right gonial and left condylar regions, while their subsequent study further confirmed additional differences involving the left gonial region [19]. In the present study, women exhibited lower mean condylar FD values than men, although the observed effect size was small to moderate. This finding is consistent with most previous studies reporting lower FD values in women than in men, suggesting relatively reduced trabecular complexity and bone density in the female mandible [13, 18-20]. However, some studies have reported no significant association between sex and FD [8, 21]. Such inconsistencies may partly be explained by methodological heterogeneity, including differences in sex distribution, sample composition, imaging protocols, and ROI selection. Overall, although the literature generally supports sex-related differences in trabecular architecture, FD alone appears to provide limited discriminative power for reliable standalone sex estimation. Since higher FD values are associated with greater trabecular complexity and increased trabeculation [21, 22], the present findings may reflect localized sex-related differences in trabecular organization, particularly in the condylar region. Masticatory loading may further explain why the condylar region exhibited the most consistent sex-related differences in the present study. Among the evaluated mandibular regions, the condyle is likely to undergo the greatest adaptive remodeling because it serves as the primary load-bearing component of the temporomandibular joint. The fibrocartilaginous surface of the mandibular condyle is specifically adapted to absorb and dissipate the substantial forces generated during mastication, thereby facilitating continuous structural remodeling in response to functional demands [23]. Therefore, the persistent significance of the condylar region in our analysis may reflect its high sensitivity to long-term biomechanical adaptation. Since males generally exert greater masticatory forces, increased mechanical loading may promote the development of a denser and more complex trabecular architecture in the male condyle, which may partly explain the higher FD values observed in males. In addition to biomechanical loading, endocrine factors may provide another plausible explanation for the observed sex-related differences. Sex steroids play a fundamental role in skeletal growth, bone metabolism, and the maintenance of bone microarchitecture throughout life [24]. Estrogen, one of the principal sex steroids, plays a critical role in preserving bone microarchitecture by suppressing osteoclast-mediated bone resorption and promoting osteogenesis, thereby maintaining trabecular connectivity and structural integrity. In contrast, estrogen deficiency, particularly during menopause, adversely affects bone microarchitecture by accelerating bone resorption and increasing trabecular deterioration [25]. Notably, the relatively narrow age range of our cohort (20–40 years) likely minimized the confounding effect of advanced age-related hormonal decline, particularly postmenopausal bone loss. Testosterone plays a crucial role in maintaining bone homeostasis throughout adulthood by interacting with androgen receptors expressed on osteoblasts, osteoclasts, osteocytes, and bone marrow stromal cells. Through these actions, it promotes bone formation while suppressing bone resorption, thereby preserving bone integrity [26]. These endocrine effects may further enhance the biomechanical adaptations observed in males, potentially contributing to the higher FD values detected in the present study. Logistic regression analysis further demonstrated that average condylar FD remained a statistically significant predictor of sex. However, the model showed limited classification performance (AUC = 0.628; accuracy = 58.5%), indicating that FD alone provides only modest predictive value, despite acceptable calibration. According to conventional ROC interpretation, this AUC reflects poor-to-fair discrimination, suggesting that the diagnostic information provided by a single imaging biomarker is inherently limited. In contrast, a previous study reported strong sex classification performance in the mandibular angle region, with an area under the ROC curve (AUC) of 0.898, indicating excellent discriminative ability [13]. This discrepancy may be attributed to differences in study population characteristics, imaging protocols, ROI selection, and sample size. Furthermore, ethnic and population-specific variations in mandibular morphology and bone remodeling patterns may also contribute to inconsistencies across studies. Therefore, FA should not be considered a standalone method for sex estimation, but rather an adjunctive tool that may complement established forensic, anthropological, radiological, and molecular identification methods. Future multimodal approaches combining FD analysis with texture-based radiomic features, machine learning algorithms, and CBCT-derived three-dimensional biomarkers may improve classification performance. Additionally, studies involving larger cohorts with external validation are needed to confirm its forensic applicability. At the population level, the generalizability of these findings should be interpreted cautiously. Sexual dimorphism in craniofacial and trabecular bone structures may vary across populations due to genetic background, ethnicity, diet, environmental influences, skeletal morphology, and functional loading patterns. Consequently, predictive thresholds derived from one population may not be directly applicable to another. Nevertheless, the biological mechanisms underlying bone remodeling and sexual dimorphism are largely universal. Therefore, FA remains a promising approach for sex estimation, provided that population-specific reference datasets and multicenter validation studies are established. From a forensic perspective, region-specific FD analysis may be particularly useful in cases involving fragmented skeletal remains, archaeological specimens, or mass-disaster victim identification, where pelvic or cranial structures are unavailable, and DNA analysis may be limited. In such scenarios, mandibular radiographic biomarkers may provide supportive identification evidence. The forensic value of FA is further supported by the relative stability of trabecular bone patterns over time. Previous investigations have demonstrated consistent FD measurements in serial panoramic radiographs obtained from the same individuals, suggesting that trabecular complexity may reflect stable structural characteristics rather than temporary imaging-related variations [27]. The present study also possesses several methodological strengths. The balanced distribution of male and female participants minimized the risk of sampling bias and enhanced the reliability of intergroup comparisons. In addition, the inclusion of bilateral ROIs from multiple mandibular sites enabled a more comprehensive assessment of trabecular organization than approaches limited to isolated anatomical regions. This multi-regional design likely increased the sensitivity for detecting localized trabecular variations that might otherwise remain unrecognized. Several limitations should be acknowledged. First, the study population consisted exclusively of individuals from a single-center Turkish cohort, which may limit the generalizability of the findings to other ethnic and geographic populations. Trabecular bone microarchitecture is influenced by multiple biological and environmental factors, including genetics, nutrition, lifestyle, and functional loading patterns. Therefore, larger multicenter investigations involving more diverse populations are necessary to validate the reproducibility and external applicability of the present findings. In addition, the inherent limitations of panoramic radiography, including image distortion, magnification, and anatomical superimposition, may have influenced trabecular assessment and FD measurements. Future studies integrating artificial intelligence-based analytical models with CBCT-derived three-dimensional trabecular assessment may further improve the diagnostic accuracy and forensic applicability of FA-based sex estimation. Moreover, establishing standardized region-specific FA protocols through large-scale multicenter investigations involving ethnically diverse populations may facilitate the integration of trabecular imaging biomarkers into forensic identification workflows.

## CONCLUSION

Within the limitations of this study, mandibular trabecular patterns assessed by FA showed region-specific sexual dimorphism, highlighting the condylar region as the most informative site for sex estimation. These findings support the potential forensic value of routinely acquired panoramic radiographs beyond conventional dental assessment. Given its accessibility, low cost, and non-invasive nature, FA may serve as a supportive tool in forensic identification workflows. However, due to population variability and the multifactorial nature of trabecular bone architecture, this method should be interpreted cautiously and in combination with other validated approaches. Future studies incorporating larger datasets and advanced predictive models are needed to enhance accuracy and clinical applicability.

## Figures and Tables

**Fig. (1):**
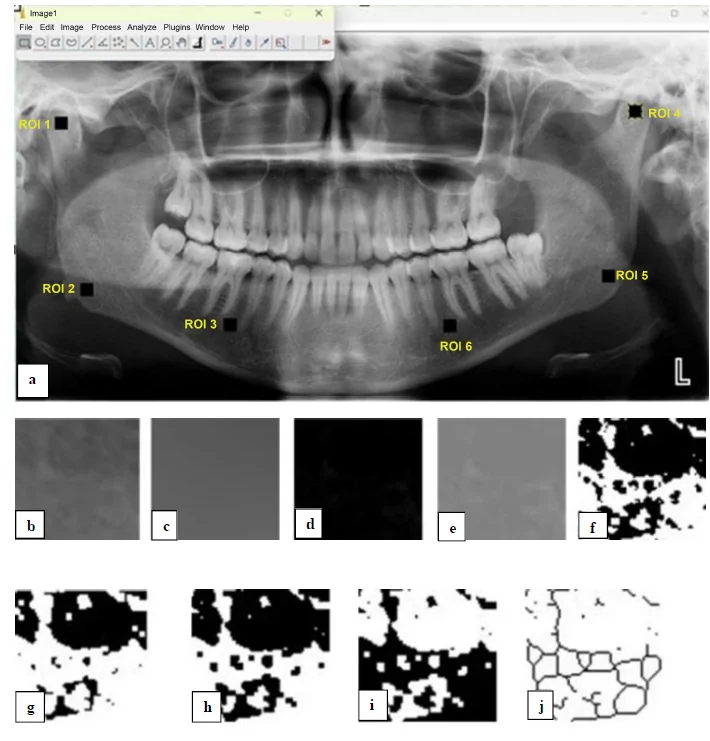
Workflow of fractal dimension analysis using ImageJ software. (**a**) ROI selection. (**b**) Duplication. (**c**) Gaussian blur (sigma = 35). (**d**) Subtraction. (**e**) Grayscale normalization (addition of 128). (**f**) Binary conversion. (**g**) Erosion. (**h**) Dilation. (**i**) Inversion. (**j**) Skeletonization.

**Table 1:** Fractal dimension values according to sex.

-	Female	Male	Test Statistics	*p*-value
Right condyle (ROI 1)	1.43 (0.99 - 1.78)	1.43 (1.12 – 1.59)	2099.500	0.954^x^
Right mandibular angle (ROI 2)	1.46 (1.11 - 1.56)	1.44 (0.96 – 1.6)	2139.500	0.902^x^
Right interdental areas (ROI 3)	1.41 (1.07 - 1.58)	1.46 (1.25 – 1.57)	1817.500	0.170^x^
Left condyle (ROI 4)	1.36 ± 0.11	1.44 ± 0.1	-4.350	**< 0.001** ^y^
Left mandibular angle (ROI 5)	1.44 (1.06 - 1.57)	1.46 (1.15 – 1.58)	1984.500	0.553^x^
Left interdental areas (ROI 6)	1.47 (1.19 - 1.58)	1.44 (1.21 – 1.53)	2538.000	**0.048** ^x^

**Note:** ^x^ Mann-Whitney U Test; ^y^ Independent two-sample t-test, mean ± standard deviation, median (minimum – maximum).

**Table 2:** Comparison of averaged bilateral fractal dimension values between females and males with effect size estimates.

Averaged Site	Female Mean ± SD	Male Mean ± SD	Mean Difference	Effect Size	*p*-value
Condyle average	1.381 ± 0.094	1.423 ± 0.091	-0.042	d = -0.45	0.012
Angle average	1.421 ± 0.096	1.424 ± 0.099	-0.003	d = -0.03	0.739
Interdental average	1.430 ± 0.074	1.430 ± 0.060	0.000	d = 0.00	0.744

**Note:** Mean difference and Cohen’s d were calculated as Female − Male. Negative values indicate lower FD values in females.

**Table 3:** Logistic regression model performance for sex prediction using average condylar fractal dimension.

Predictor	OR per 0.1 FD Increase	95% CI	*p*-value	LR test *p*-value	McFadden R^2^	AUC	Accuracy	Sensitivity	Specificity	Hosmer–Lemeshow p
Average condyle FD	0.61	0.41–0.91	0.014	0.011	0.036	0.628	58.5%	53.8%	63.1%	0.692

## Data Availability

The Dicle University Faculty of Dentistry provides the data for the study; however, it is not publicly accessible due to legal protections. Nevertheless, data are accessible upon a reasonable request from the corresponding author [S.A.B].

## LIST OF ABBREVIATIONS

FA: Fractal Analysis
FD: Fractal dimension
ROI: Region of interest
ICC: Intraclass correlation coefficient
OR: Odds ratio
CI: Confidence interval

## STANDARDS OF REPORTING

STROBE guidelines were followed.
